# Adeno-associated virus 9 vector-mediated cardiac-selective expression of human secretory leukocyte protease inhibitor attenuates myocardial ischemia/reperfusion injury

**DOI:** 10.3389/fcvm.2022.976083

**Published:** 2022-08-19

**Authors:** Podsawee Mongkolpathumrat, Nitirut Nernpermpisooth, Anusak Kijtawornrat, Faprathan Pikwong, Wannapat Chouyratchakarn, Rungrueang Yodsheewan, Sasimanas Unajak, Sarawut Kumphune

**Affiliations:** ^1^Biomedical Engineering Institute (BMEI), Chiang Mai University, Chiang Mai, Thailand; ^2^Integrative Biomedical Research Unit (IBRU), Faculty of Allied Health Sciences, Naresuan University, Phitsanulok, Thailand; ^3^Department of Cardio-Thoracic Technology, Faculty of Allied Health Sciences, Naresuan University, Phitsanulok, Thailand; ^4^Department of Physiology, Faculty of Veterinary Science, Chulalongkorn University, Bangkok, Thailand; ^5^Department of Pathology, Faculty of Veterinary Medicine, Kasetsart University, Bangkok, Thailand; ^6^Department of Biochemistry, Faculty of Science, Kasetsart University, Bangkok, Thailand

**Keywords:** ischemic/reperfusion injury, secretory leukocyte protease inhibitor (SLPI), gene therapy, cardiac selective expression, cardioprotection

## Abstract

Protease enzymes contribute to the initiation of cardiac remodeling and heart failure after myocardial ischemic/reperfusion (I/R) injury. Protease inhibitors attenuate protease activity and limit left ventricular dysfunction and remodeling. Previous studies showed the cardioprotective effect of secretory leukocyte protease inhibitor (SLPI) against I/R injury. However, overexpression of SLPI gene in cardiovascular diseases has only been investigated in an *in vitro* experiment. Here, cardiac-selective expression of the human secretory leukocyte protease inhibitor (hSLPI) gene and its effect on I/R injury were investigated. Adeno-associated virus (AAV) serotype 9 carrying hSLPI under the control of cardiac-selective expression promoter (cardiac troponin, cTn) was intravenously administered to Sprague–Dawley rats for 4 weeks prior to coronary artery ligation. The results showed that myocardial-selective expression of hSLPI significantly reduced infarct size, cardiac troponin I (cTnI), creatine kinase-MB (CK-MB), and myoglobin levels that all served to improve cardiac function. Moreover, overexpression of hSLPI showed a reduction in inflammatory cytokines, oxidatively modified protein carbonyl (PC) content, ischemia-modified albumin (IMA), and necrosis and cardiac tissue degeneration. In conclusion, this is the first study to demonstrate cardiac-selective gene delivery of hSLPI providing cardioprotection against myocardial I/R injury in an *in vivo* model.

## Introduction

Cardiovascular diseases (CVDs), particularly ischemic heart disease, are the most common cause of global deaths (1). During myocardial ischemia and reperfusion, various biochemical processes including post-ischemic inflammation, production of oxygen radicals, polymorphonuclear cell infiltration, and protease release occur. These contribute to tissue damage, cell necrosis, and subsequent functional impairment (2, 3). Many reports show that protease enzymes, including calpain–cathepsin and matrix metalloproteinase (MMP), contribute to the initiation of cardiac remodeling and heart failure after ischemia/reperfusion (I/R) injury (4–6). Protease inhibitors are potential candidates that attenuate the activity of activated protease enzymes and progression of post-ischemic left ventricular dysfunction and remodeling (7–9). Many synthetic protease enzyme inhibitors have been developed; however, these present off-targeting effects as serious concerns for adverse outcomes (10). Therefore, mediated cardiac-selective expression of a protease inhibitor protein that is normally expressed in the body could be a promising candidate for a safe and effective novel treatment.

Secretory leukocyte protease inhibitor (SLPI) is an 11.7-kDa serine protease inhibitor that is expressed in various cell types, especially epithelial cells in mucous areas (11). A previous report showed low expression of SLPI in a rat cardiac myoblast (H9c2) cell line (12) and adult heart tissue, while SLPI showed a cardioprotective effect in both *in vitro* and *ex vivo* models against I/R and hypoxia/reperfusion (H/R) injury with reduced intracellular reactive oxygen species (ROS) levels, cell injury, and increased cell viability (12–14). A recent *in vivo* study of SLPI also showed reduced infarct size and improved cardiac function after intravenous administration of recombinant hSLPI protein at the onset of reperfusion in a left anterior descending artery (LAD) ligation model (15). Our previous findings showed the cardioprotective effect of recombinant hSLPI against myocardial I/R injury in several study models. However, overexpression of SLPI in cardiovascular diseases has only been investigated in an *in vitro* experiment (12). One of the clinical limitations of using recombinant hSLPI as a therapeutic agent is its short half-life in circulation (16), while it can also be inactivated by enzymes that are secreted from the respiratory tract such as cathepsin (17). Therefore, tissue overexpression of SLPI could provide beneficial effects on target tissue rather than exogenous administration.

Gene therapy has been demonstrated as a novel efficient approach in preclinical studies (18, 19). Invasive interventions such as PCI and CABG may not be suitable for every patient, especially the elderly and those with multiple comorbidities. To complement heart-healthy lifestyles and pharmacotherapy, gene therapy is now emerging as a therapeutic option and an active exploration area providing hope for patients (20). Targeting gene delivery to induce interesting protein overexpression in the heart is applicable to a broad range of CVDs (21). The cardiovascular approach uses the adeno-associated virus (AAV) vector for a gene delivery vector to provide long-term transgene overexpression, especially AAV serotype 9, (AAV9) (22). However, overexpression of SLPI using cardiac-selective AAV9-SLPI in myocardial I/R injury has not been investigated.

This study demonstrated AAV9-SLPI with a cardiac-selective promoter for the first time as a therapeutic vector for the attenuation of myocardial I/R injury. The results suggested a novel modality for overexpression of the protease inhibitor using cardiac-selective AAV9-SLPI as a potential therapeutic strategy for ischemic heart disease.

## Materials and methods

### Adeno-associated virus particle

Viral particles for pseudotyped AAV serotype 9 containing the transgene for the human secretory leukocyte protease inhibitor (hSLPI) (gene ID number: 6590, NCBI's Reference Sequence: NM_003064.4, accession number NM_003064) were customized and produced from Genemed Biotechnology (Shanghai, China). The AAV vectors carrying human SLPI cDNA are controlled for gene expression under the control of cardiac-selective (cardiac troponin, cTn) promoter (AAV9-cTnT-hSLPI). The control rAAV9 virus was generated without hSLPI gene. Purification and validation were performed by the company prior to delivery.

### Experimental animal ethical approval

Male Sprague–Dawley rats were purchased from Nomura Siam International, Bangkok, Thailand. All care and use of the animal subjects followed the ARRIVE guidelines and the Guidance on the Operation of the Animals (Scientific Procedures) Act 1986 and the World Health Organization Guidelines for Breeding and Care of Laboratory Animals. All animal protocols were approved by the Institutional Animal Care and Use Committee, Chulalongkorn University Laboratory Animal Centre (CULAC, protocol no. 1973025). The experiments were followed by the animal research guideline to minimize the pain, suffering, and distress.

Twenty-one adult male Sprague–Dawley rats (8 weeks old) weighing between 250 and 300 g (*n* = 7 per group) were purchased from Nomura Siam International, Bangkok, Thailand. All animals were maintained in an environmentally controlled condition (22°C ± 1°C, RH 50 ± 10%, 12:12-h light/dark cycle) at the Chulalongkorn University Laboratory Animal Centre (CULAC), Bangkok, Thailand.

### Experimental groups

A total of 39 adult male rats were used in this study. The rats were systemically injected with 0.5 x 10^12^ pseudotyped viral particles *via* the intravenous route. For IV injection, the rats were anesthetized with 1.8–2.0% isoflurane in oxygen, while viral solution was slowly injected *via* the jugular vein. For determining the successful SLPI overexpression and toxicity of AAV gene delivery, AAV9 and AAV9-SLPI (*n* = 3/group) were injected for 4 weeks, and then, several organs including heart, brain, muscle, liver, kidney, lung, spleen, and serum were collected. The expression of SLPI was performed by qRT-PCR and ELISA, while the toxicity of AAV gene delivery was performed by determining body weight, heart weight, and serum biomarkers.

For I/R injury, the rats were randomly divided into three groups (*n* = 11/group) including rats receiving sham operation (sham) group, rats receiving control viral particle injection (AAV9) group, and rats receiving AAV9 viral particle carrying human SLPI cDNA injection (AAV9-SLPI) group. The rats were received AAV9 or AAV9-SLPI for 4 weeks before proceeding to myocardial I/R injury. The surgery for myocardial I/R injury by left anterior descending (LAD) coronary artery ligation was performed in all animal groups. In sham group, the rats were performed a surgery without ligation. In AAV9 and AAV9-SLPI group, the rats were subjected to LAD ligation for 30 mins and released a ligation to perform the reperfusion for 120 mins.

### Animal procedures of myocardial I/R model in rats

Animal surgical preparation was performed as described previously (15). In brief, LAD ligation was performed for inducing ischemia for 30 mins, and the ligation was released for reperfusion for 120 mins. Then, the hearts were quickly excised for infarct size measurement and a blood sample was collected for the rats. Throughout the surgery, ECG and LVP were measured and recorded by an IOX Data Acquisition System version 2.10.8.6 (EMKA Technologies, Paris, France). All ECG and LVP data were analyzed by ECG Auto software version 3.5.5.12 (EMKA Technologies, Paris, France). The LVP parameters that were analyzed consist of heart rate (HR), end-diastolic pressure (EDP), end-systolic pressure (ESP), developed pressure (devP), maximum rate of rise in the LVP (dP/dt _max_), minimum rate of fall in the LVP (dP/dt _min_), contractility index (CI), and tau.

### Evaluation of infarct size

The evaluation of infract size and area at risk protocol was performed as described in the previous study (15). The hearts were quickly excised from the rats at the end of the protocol. The hearts were weighted and re-ligated at the LAD, which was a place of ligation. The hearts were injected with 2% (w/v) phthalo blue in PBS for differentiating the area at risk (a non-perfusion area during ligation) from a non-ischemic area. After phthalo blue perfusion, the hearts were transversally sliced into 1-mm-thick slices and the slices were incubated in 1% (w/v) 2,3,5-triphenyl tetrazolium chloride (TTC) at 37°C for 10 mins to define the necrotic myocardium. The contrast between stained and unstained TTC areas was enhanced by incubating in 10% formalin (v/v) for 15–20 h. An infarct area (TTC-negative) and a non-ischemic area (phthalo blue-stained area) were evaluated by using ImageJ software.

### Determination of serum biomarkers

Blood was rapidly collected from the rats at the end of experiments into Becton–Dickinson Company (BD) Vacutainer®. The collected blood was centrifuged at 3,000 × g for 10 mins at 4°C followed by serum collection that was stored at −8°C until analysis. The serum was thawed, and the measured serum biomarkers including cardiac troponin I (cTnI), creatine kinase (MB isoenzyme, CK-MB), and myoglobin were also analyzed by using an automated biochemistry analyzer (Cobas c 111 analyzers, Roche, Basel, Switzerland). In addition, liver function and renal function test markers including alanine aminotransferase (ALT), aspartate aminotransferase (AST), blood urea nitrogen (BUN), and creatinine were also determined.

### Tissue homogenate and protein collection

At the end of I/R injury condition, the ventricular tissue was rapidly collected for each analysis, quickly frozen in liquid nitrogen, and stored at −80°C. One hundred milligrams of each organ was selected, mixed with lysis buffer (20 mM Tris–HCl pH 6.8, 1 mM Na_3_VO_4_, 5 mM NaF, and cOmplete™ protease inhibitor cocktail), and homogenized with a hand homogenizer on ice. The extracted tissue was centrifuged at 14,000 rpm for 10 mins at 4°C, and the supernatant was collected and stored at −80°C until further experiments.

### Determination of *slpi* expression by qRT-PCR

Total RNA was extracted from frozen tissues, and cDNA synthesis and quantitative reverse-transcription PCR were performed as described previously (23). The primers were designed from a coding sequence of SLPI (GenBank locus: NM_003055)—SLPI forward primer: AGC GTG ACT TGA AGT GTT GCA TG and reverse primer: GAA AGG ACC TGG ACC ACA CAG A. A relative gene expression level was analyzed and calculated by normalizing the differences in cycle threshold number (*C*_*T*_) with an internal control reference gene, which is glyceraldehyde-3-phosphate dehydrogenase (GAPDH) (24). The relative gene expression level was calculated following the ΔΔ*C*_*T*_ method with Bio-Rad® CFX Manager™ 3.1 software. The gene expression level of hSLPI in AAV9-SLPI was determined as the relative expression (fold change), which was presented as the ratio of SLPI gene expression in different tissues from the rats receiving AAV9-SLPI in relative to the rats receiving AAV9 control viral particle.

### Determination of SLPI level by ELISA

A SLPI expression level was determined with the Human SLPI (secretory leukocyte protease inhibitor) ELISA Kit (Elabscience, Texas, USA) as described previously (13). All reagents were prepared at room temperature before use. The protein samples were measured by the Bradford assay, and then, 100 μg of extracted protein was added into the well and incubated for 90 mins at 37°C. After incubation, the liquid was decanted from each well-without washing. Biotinylated Detection Ab working solution was added into the well and incubated for 1 h at 37°C. Then, all solutions were decanted and wash buffer was added into each well. The wells were washed by soaking in 350 μl of wash buffer for 1 min and decanting the wash buffer. The plates were inverted to remove the liquid and drained on the clean absorbent paper. The plates were washed three times, and 100 μl of HRP-conjugated working solution was added into each well. After incubation for 30 mins at 37°C, the liquid was decanted from the plate and the washing process was repeated five times. After being washed, 90 μl of substrate reagent was added into the wells and incubated for 15 mins at 37°C. In this step, the plate was protected from the light. Then, 50 μl of stop solution was added into the well. The optical density (O.D.) was measured by a microplate reader at 450 nm and expressed in ng/mg of total protein.

### Determination of inflammatory cytokine level

Inflammatory cytokine levels were determined by using the ABTS ELISA Buffer Kit, and PeproTech® was performed as described previously (23). All solutions of ELISA were gently prepared at room temperature. For antibody coating on microplate, 100 μl of 1 μg/ml capture antibody was added into a 96-well plate and incubated overnight at room temperature. The capture antibody was removed and blotted on a paper towel. Each well-was washed four times with 200 μl of washing buffer solution. After washing, 200 μl of blocking solution was added into the wells and incubated for 1 h at room temperature. A washing step was performed again four times. The protein samples were measured by the Bradford assay. Then, 100 μg of serum protein and the extracted cardiac protein was added into each well and incubated for at least 2 h at room temperature. The plates were washed again four times. After that, 100 μl of detected antibody was added into the well and incubated for 2 h. The plates were washed again four times. One hundred microliter of avidin–HRP-conjugated antibody (1:2,000) was added into the well and incubated at room temperature for color development. Inflammatory cytokine-level absorbance was measured by a spectrophotometer at 405 nm with wavelength correction at 650 nm and expressed in ng/mg of total protein.

### Determination of oxidatively modified protein: Protein carbonyl content and ischemia-modified albumin

Protein carbonyl (PC) content level and ischemia-modified albumin (IMA) were determined by the colorimetric DNPH assay and albumin–cobalt binding (ACB) assay, respectively, as previously described (25, 26). 2,4-dinitrophenylhydrazine (DNPH) spectrophotometric assay, blood serum, and extracted cardiac protein were thawed from the storage and diluted at 1:10 with phosphate-buffered saline (PBS). Then, 200 μl of diluted protein samples was added into 800 μl of 10 mM DNPH in 2.5 M HCl. The samples were precipitated by adding 1 ml 20% (w/v) trichloroacetic acid (TCA) and then centrifuged at 10,000 × g for 10 mins at 4°C. The pellets were washed three times with 1 ml of 1:1 (v/v) ethanol/ethyl acetate and centrifuged at 10,000 × g for 10 mins at 4°C. After washing, the pellets were resuspended in 500 μl 6 M guanidine hydrochloride and centrifuged at 10,000 × g for 10 mins at 4°C. The supernatant was collected, and the absorbance was measured at 370 nm. PC content (nmol/mg) was calculated by following the formula from the previous study (27) by using 6 M guanidine hydrochloride as a blank.

In the albumin–cobalt binding (ACB) assay, the reaction was prepared by gently mixing 200 μl of serum or extracted cardiac protein and 50 μl of 0.1% (w/v) cobalt chloride. The medium was incubated for 10 mins at room temperature for adequate cobalt–albumin binding. A colored reaction was performed by adding 50 μl 1.5 mg/ml dithiothreitol (DTT) into the medium and incubating for 2 mins at room temperature. The colored reaction was quenched by adding 1 ml sodium chloride (NaCl) into the medium. Ischemia-modified albumin (IMA) was determined by measuring absorbance at 470 nm. The IMA level was calculated by using a serum and extracted cardiac protein blank without DTT as a blank of individual samples.

### Histopathology

Each heart was sliced into four equal transverse (coronal) slices and cassetted and fixed directly in 10% buffered formalin for histopathological process. In brief, the slices were dehydrated, embedded into paraffin, and cut into 5–10-μm-thick sections (PFM Rotary 3005E, Germany). Then, the sections were stained with hematoxylin and eosin (H&E) for histopathological analysis. All histopathological examinations were performed by an experienced pathologist. The pathologist was blind toward the experiment group, and the examination and scoring were performed under a light microscope (Olympus, Tokyo, Japan). Myocardial I/R injury was histopathologically assessed and scored according to the previous report (28) using the published morphological criteria: 0, no damage; 1 (mild), interstitial edema and localized necrosis; 2 (moderate), widespread myocardial cell swelling and necrosis; 3 (severe), necrosis with contraction bands and compressed capillaries; or 4 (highly severe), diffuse necrosis with contraction bands, compressed capillaries, and hemorrhage.

### Statistical analysis

All values were expressed as mean ± standard deviation (S.D.). The normal distribution of the data was analyzed by the Shapiro–Wilk test. All comparisons involving more than one group were assessed for significance using one-way analysis of variance (ANOVA), followed, when appropriate, by the Tukey–Kramer test. A *p*-value of <0.05 was considered significant.

## Results

### Effect of an *in vivo* safety of adeno-associated viral gene delivery of SLPI on animal body weight and blood chemistry

**Figure 1:**
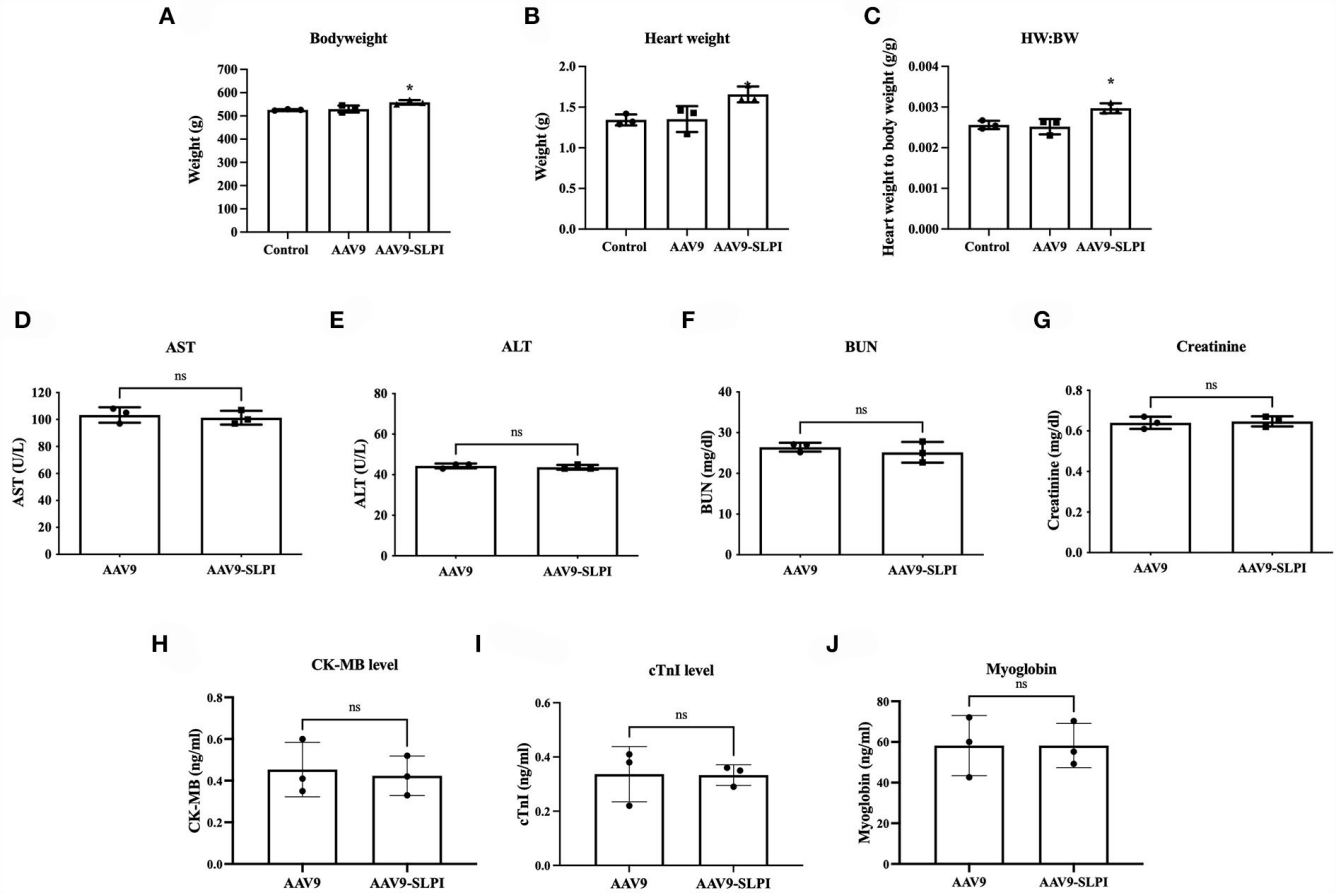
Determination of body weight of experimental animal after viral treatment in I/R injury **(A)**, heart weight **(B)**, heart weight-to-body weight ratio **(C)**. Determination of serum toxicity biomarkers after adeno-associated viral particles injection including AST **(D)**, ALT **(E)**, BUN **(F)**, and creatinine **(G)**. Determination of serum myocardium injury biomarkers after AAV9 injection including CK-MB **(H)**, cTnI **(I)**, and myoglobin **(J)** (*p < 0.05 *vs*. control).

Animal body weight and heart weight were measured at the end of the protocol. The results showed that the body weight of rat receiving AAV9 control viral particle (AAV9 groups) was not significantly different when compared with the non-viral injected (control group) (532.3 ± 15.01 g vs. 525.7 ± 3.0 g, *p* > 0.05). In contrast, the body weight of rat receiving AAV9-SLPI viral particle (AAV9-SLPI group) was significantly different when compared with AAV9 group (560.0 ± 9.73 g vs. 532.3 ± 15.01 g, *p* < 0.05). Similarly, the heart weight of AAV9 group was not significantly different when compared with control group (1.44 ± 0.15 g vs. 1.35 ± 0.06 g, *p* > 0.05), but significantly different when compared with AAV9-SLPI group (1.44 ± 0.15 g vs. 1.60 ± 0.09 g, *p* < 0.05) (Figures 1A,B). Then, the heart weight-to-body weight ratio was calculated, which showed a significant difference in AAV9-SLPI group compared with AAV9 group (0.0028 ± 0.0001 g vs. 0.0027 ± 0.0001, *p* < 0.05) (Figure 1C).

For determining the toxicity of SLPI gene delivery, the serum biomarkers for liver and renal toxicity including AST, ALT, BUN, and creatinine were determined and showed no significant difference between AAV9 and AAV9-SLPI groups (AST: 103.3 ± 5.68 U/L vs.101.3 ± 5.13 U/L, ALT: 44.33 ± 1.15 U/L vs. 43.67 ± 1.15 U/L, BUN: 26.34 ± 1.06 U/L *vs*. 25.17 ± 2.55 U/L, creatinine: 0.64 ± 0.03 U/L *vs*. 0.64 ± 0.02 U/L, *p* > 0.05) (Figures 1D–G). Moreover, the serum biomarkers for myocardium injury including CK-MB, cTnI, and myoglobin were also determined and showed no significant difference between AAV9 and AAV9-SLPI groups (CK-MB: 0.45 ± 0.13 vs. 0.42 ± 0.09 ng/ml, cTnI: 0.33 ± 0.10 vs. 0.33 ± 0.03, myoglobin: 58.23 ± 14.83 vs. 58.23 ± 10.87) (Figures 1H–J).

### Expression of *slpi* gene and SLPI protein

**Figure 2:**
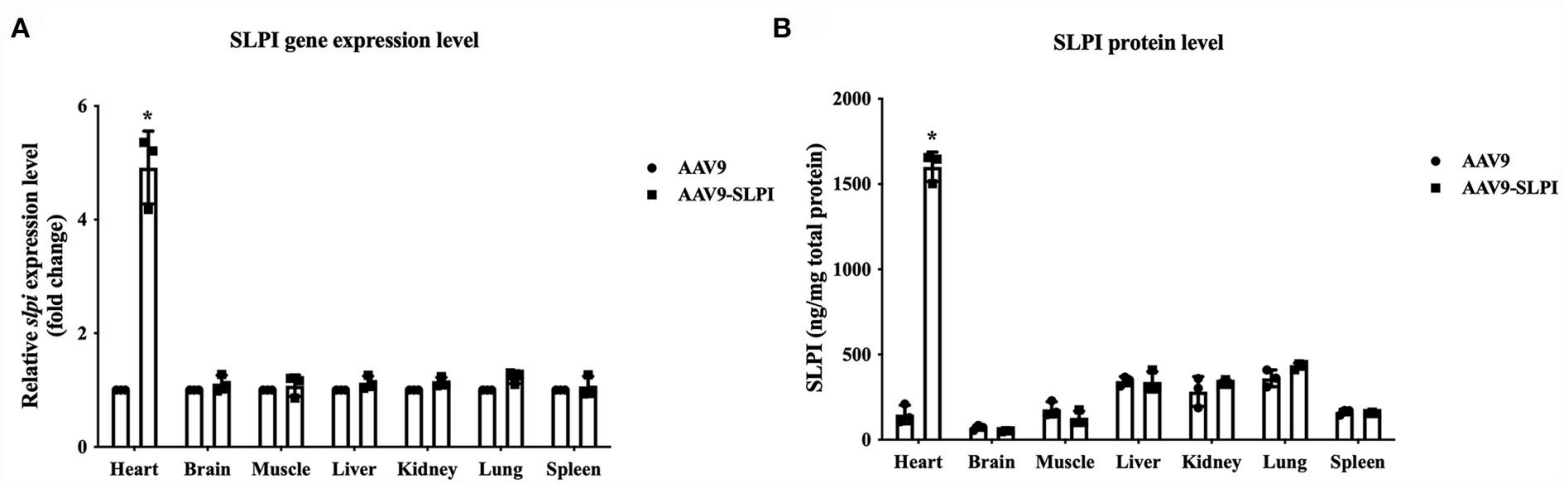
Determination of *slpi* gene **(A)** and protein expression level **(B)** in organs of I/R injury rats treated with viral particles (*p < 0.05 vs. AAV9).

After infection with AAV9 control viral particle and AAV9-SLPI viral particle, several organs including heart, brain, muscle, liver, kidney, lung, and spleen were collected and measured for *slpi* expression and SLPI protein level. The results showed that the *slpi* expression level from the heart tissue of AAV9-SLPI group was significantly higher than that of AAV9 group (4.917 ± 0.64 vs. 1 ± 0.00, *p* < 0.05). Similarly, the SLPI protein level from the heart tissue of AAV9-SLPI group was significantly higher than that of AAV9 group (1601.59 ± 85.97 ng/mg vs. 148.09 ± 54.18 ng/mg, *p* < 0.05). However, the *slpi* and protein expression levels were not significantly different between groups of each organ (Figure 2).

### AAV9-mediated SLPI expression reduced infarct size and cardiac injury markers in I/R rats

**Figure 3:**
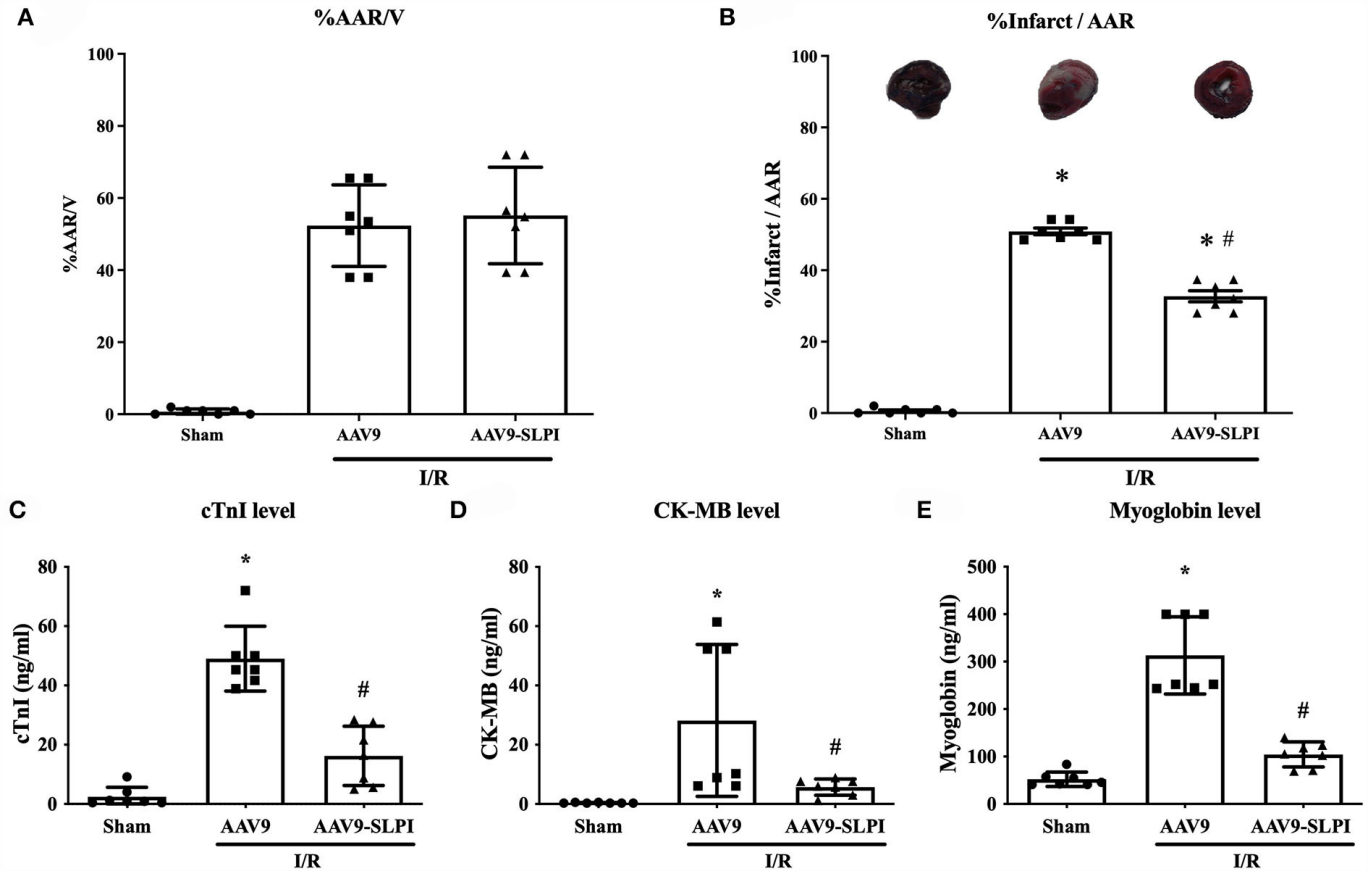
Determination of infarct size **(A)**, area at risk **(B)**, and serum cardiac biomarkers including cTnI **(C)**, CK-MB **(D)**, and myoglobin **(E)** in I/R rats (*p < 0.05 vs. sham) (^#^*p* < 0.05 vs. AAV9).

The results showed that the percentage of area at risk (AAR) to ventricle between AAV9 and AAV9-SLPI groups was not significantly different (52.60 ± 11.31 vs. 54.96 ± 13.39, *p* > 0.05) (Figure 3A). For infarct size measurement, the results showed that the percentage of infarct size per area at risk (%infarct/AAR) was significantly increased in AAV9 group when compared with sham group (50.68 ± 2.45 *vs*. 0.6 ± 0.78, *p* < 0.05) (Figure 3B), whereas the infarct size of AAV9-SLPI group was significantly lower when compared with AAV9 group in I/R rats (32.69 ± 4.09 vs. 50.68 ± 2.45, *p* < 0.05) (Figure 3B). The results of determination of serum cardiac injury biomarkers including cTnI, CK-MB, and myoglobin showed that all biomarkers were significantly higher in the rats subjected to I/R (AAV9, AAV9-SLPI, vs sham) (Figures 3C–E). In contrast, there was a significantly lower cardiac biomarker level in AAV9-SLPI group than that in AAV9 group (cTnI: 16.24 ± 10.02 ng/ml vs. 49.58 ± 10.90 ng/ml, CK-MB: 5.72 ± 2.73 ng/ml vs. 27.79 ± 25.61 ng/ml, myoglobin: 104.4 ± 26.40 ng/ml *vs*. 307.9 ± 81.36 ng/ml, *p* < 0.05) (Figures 3C–E).

### AAV9-mediated SLPI expression improved left ventricular pressure parameters

The LVP parameters were evaluated at the end of the protocol by pressure catheters in the left ventricle of I/R rats after treating with viral particles. ECG and LVP parameters include HR, EDP, ESP, devP, dP/dt_max_, dP/dt_min_, CI, and tau. The results showed that all baseline LVP parameters were not significantly different among groups (Table 1). The HR was not significantly different among groups (Table 1). I/R injury showed significantly increased EDP, dP/dt_min_, and tau and reduced ESP, devP, dP/dt_max_, and CI in AAV9 group (Table 2), while the AAV9-mediated SLPI expression in AAV9-SLPI showed significantly reduced EDP and dP/dt_min_ and increased ESP, devP, dP/dt_max_, and CI (Table 2).

**Table 1:** Baseline LVP parameters.

	**Group**		
**Parameters**	**Control** **(*n* = 7)**	**AAV9** **(*n* = 7)**	**AAV9-SLPI** **(*n* = 7)**
EDP (mmHg)	5.07 ± 0.51	4.77 ± 1.05	4.82 ± 1.33
ESP (mmHg)	102.9 ± 17.68	110.2 ± 9.99	101.1 ± 13.13
dP/dt_max_ (mmHg/s)	5,360 ± 702.5	5,092 ± 972.4	5,435 ± 911.5
CI	109.8 ± 5.83	109.8 ± 8.37	108.0 ± 7.88
dP/dt_min_ (mmHg/s)	−4,281 ± 782.7	−4,178 ± 884.1	−4,008± 781.8
Tau/e (ms)	10.75 ± 1.60	10.67 ± 1.57	11.38 ± 1.46
devP (mmHg)	107.1 ± 13.44	103.5 ± 10.98	107.6 ± 14.22
HR (bpm)	298.9 ± 24.86	311.6 ± 40.12	295.7 ± 12.18

**Table 2:** Effect of AAV9-SLPI on LVP parameters in I/R rats at the end of reperfusion period.

	**Group**		
**Parameters**	**Control** **(*n* = 7)**	**AAV9** **(*n* = 7)**	**AAV9-SLPI** **(*n* = 7)**
EDP (mmHg)	4.05 ± 0.98	8.67 ± 0.77^*^	5.95 ± 1.36^#^
ESP (mmHg)	106.2 ± 3.34	91.81 ± 6.13^*^	115.8 ± 1.41^#^
dP/dt_max_ (mmHg/s)	5,265 ± 322.1	4,173 ± 414.3^*^	4,954 ± 100.4^#^
CI	105.9 ± 6.26	95.77 ± 0.35^*^	104.0 ± 3.65^#^
dP/dt_min_ (mmHg/s)	−4,578 ± 215.3	−3,160 ± 107.3^*^	−4,662± 154.6^#^
Tau/e (ms)	10.78 ± 0.82	12.94 ± 1.00^*^	11.75 ± 0.44
devP (mmHg)	109.5 ± 3.60	93.44 ± 1.162^*^	108.9 ± 6.61^#^
HR (bpm)	297.8 ± 30.78	295.9 ± 18.27	297.4 ± 18.26

(* p < 0.05 vs. sham) (

# p < 0.05 vs. AAV9).

### AAV9-mediated SLPI expression reduced inflammation in I/R rats

**Figure 4:**
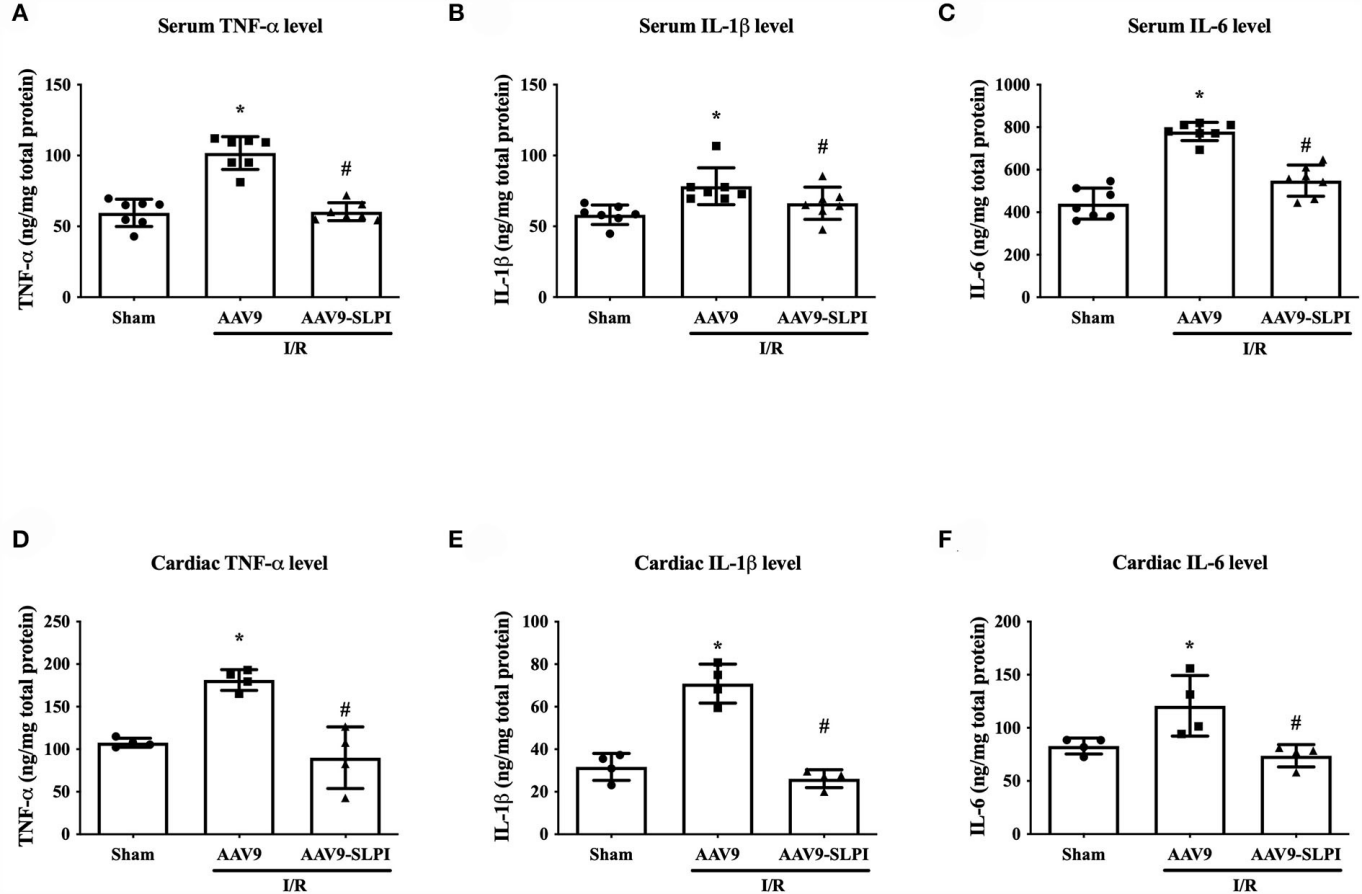
Determination of inflammatory cytokines after treating with viral particles including serum TNF-α level **(A)**, IL-1β level **(B)**, IL-6 **(C)**, and cardiac TNF-α level **(D)**, IL-1β level **(E)**, and IL-6 **(F)** on I/R injury rats (*p < 0.05 vs. sham) (^#^*p* < 0.05 vs. AAV9).

In this study, the inflammatory cytokines including TNF-α, IL-1β, and IL-6 were measured for both blood serum and heart tissue extraction. The results showed that all serum inflammatory cytokine levels were significantly increased in myocardial I/R (AAV9 group) when compared with sham group (TNF-α: 101.6 ± 11.57 ng/mg vs. 59.55 ± 9.58 ng/mg, *p* < 0.05, IL-1β: 80.20 ± 12.97 ng/mg vs. 58.20 ± 6.92 ng/mg, *p* < 0.05, and IL-6: 775.1 ± 42.92 ng/mg vs. 440.7 ± 72.76 ng/mg, *p* < 0.05) (Figures 4A–C). In contrast, the inflammatory cytokine levels in AAV9-mediated SLPI expression (AAV9-SLPI group), subjected to myocardial I/R, were significantly lower when compared with AAV9 group (TNF-α: 60.35 ± 6.34 ng/mg vs. 101.6 ± 11.57 ng/mg, *p* < 0.05, IL-1β: 66.34 ± 11.37 ng/mg vs. 80.20 ± 12.97 ng/mg, *p* < 0.05, and IL-6: 548.4 ± 73.39 ng/mg vs. 775.1 ± 42.92 ng/mg, *p* < 0.05) (Figures 4A–C).

In cardiac inflammatory cytokine levels, myocardial I/R in AAV9 group was significantly higher than in sham group (TNF-α: 186.1 ± 12.17 ng/mg vs. 108.2 ± 5.38 ng/mg, *p* < 0.05, IL-1β: 29.48 ± 6.32 ng/mg vs. 69.03 ± 9.17 ng/mg, *p* < 0.05, and IL-6:113.1 ± 28.43 ng/mg vs. 81.31 ± 7.51 ng/mg, *p* < 0.05) (Figures 4D–F). The AAV9-SLPI infection has significantly reduced inflammatory cytokine levels when compared with AAV9 group (TNF-α: 85.92 ± 36.24 ng/mg vs. 186.1 ± 12.17 ng/mg, *p* < 0.05, IL-1β: 29.48 ± 4.20 ng/mg vs. 69.03 ± 9.17 ng/mg, *p* < 0.05, and IL-6: 74.23 ± 10.50 ng/mg vs. 113.1 ± 28.43 ng/mg, *p* < 0.05) (Figures 4A–C).

### Overexpression of SLPI reduced I/R-induced cardiac tissue degeneration

**Figure 5:**
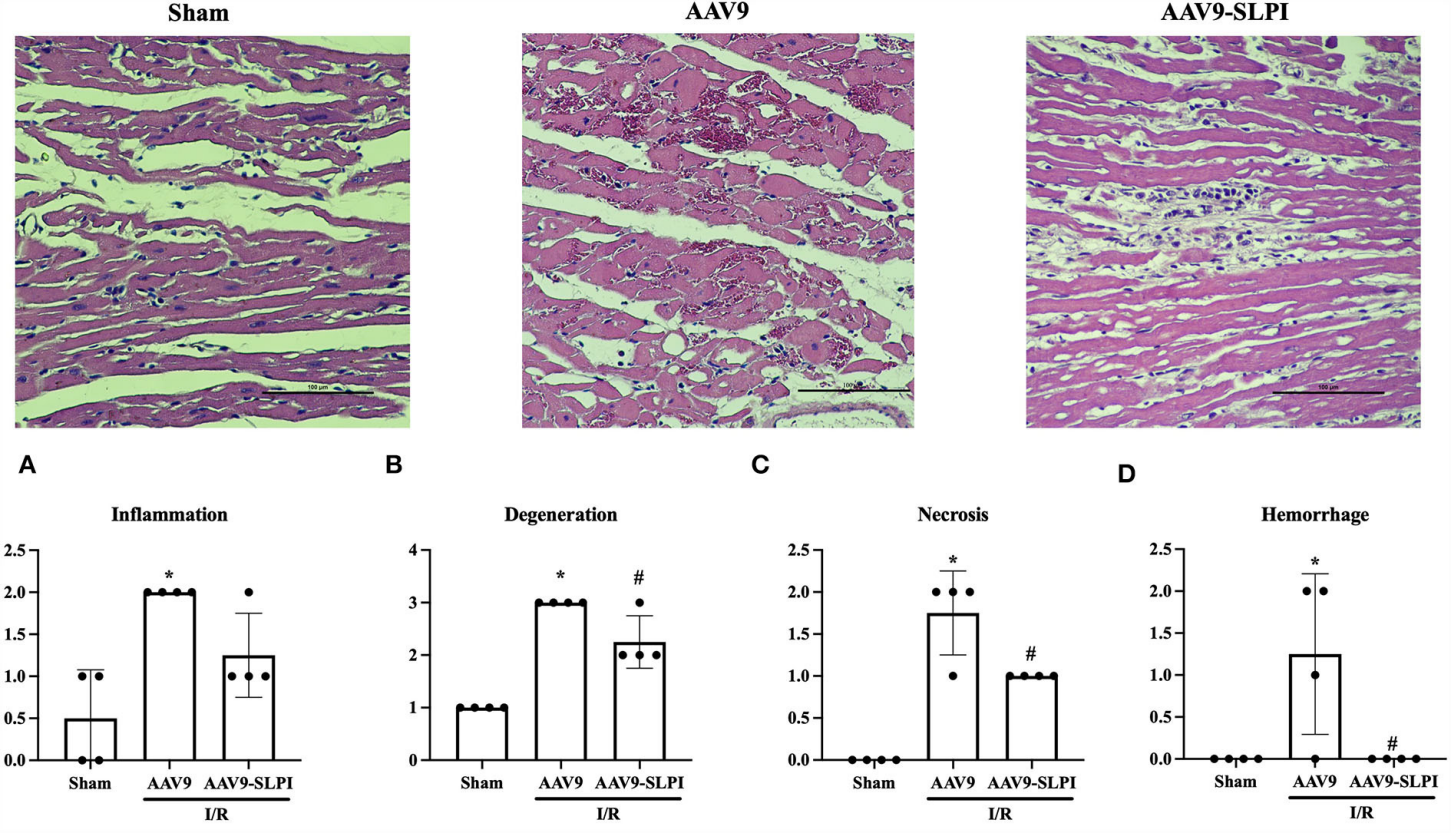
Myocardial histology parameters consists of inflammation **(A)**, degeneration **(B)**, necrosis **(C)**, and hemorrhage **(D)** (*p < 0.05 *vs*. sham) (^#^*p* < 0.05 *vs*. AAV9).

Histopathology was performed in myocardial tissue that overexpresses SLPI by using cardiac-selective AAV9 delivery. To determine the severity of cardiac tissue histopathology, severity of inflammation, tissue degeneration, necrosis, and hemorrhage was graded on a scale ranging from 0 to 2 (Figure 5).

The results showed that I/R injury caused inflammation, tissue regeneration, necrosis, and hemorrhage in cardiac tissue (Figures 5A–D). Overexpression of SLPI significantly reduced tissue regeneration necrosis and hemorrhage (Figures 5B–D). Although the tissue inflammation was not significantly reduced by SLPI overexpression, a trend of reduction could be observed (Figure 5A).

### AAV9-mediated SLPI expression reduced serum and tissue oxidatively modified protein in I/R rats

**Figure 6:**
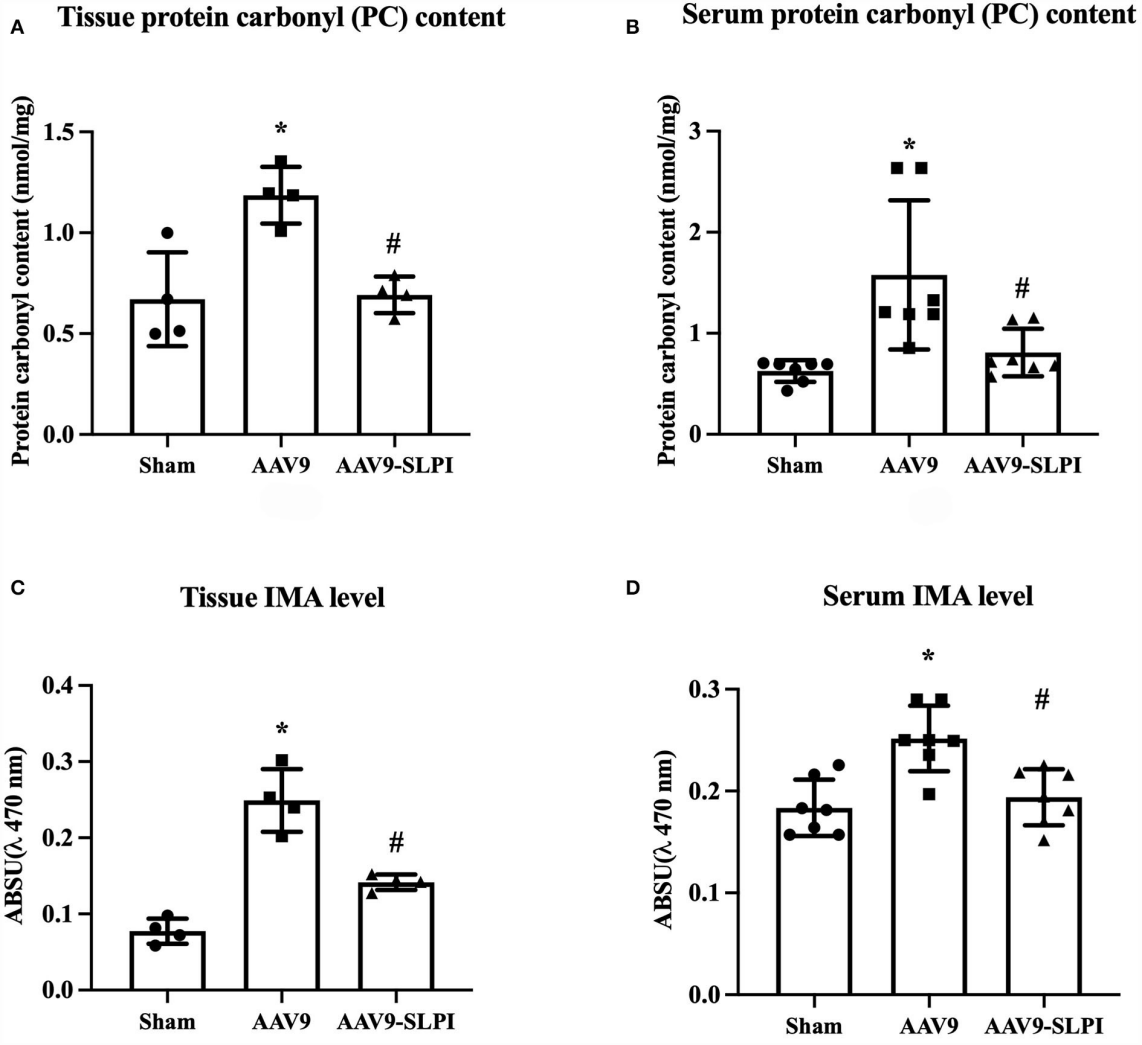
Determination of oxidatively modified cardiac protein level including tissue PC content level **(A)**, serum PC content level **(B)**, tissue IMA level **(C)**, and serum IMA level **(D)** (*p < 0.05 *vs*. sham) (^#^*p* < 0.05 *vs*. AAV9).

The protein carbonyl (PC) content level and Ischemia-modified albumin (IMA) of serum and cardiac tissue were measured. The results showed that there was a significant increase in both serum and tissue PC content levels in AAV9 subjected to I/R injury when compared with sham group (tissue: 1.18 ± 0.14 nmol/mg *vs*. 0.67 ± 0.23 nmol/mg, *p* < 0.05, Serum: 1.44 ± 0.73 nmol/mg vs. 0.61 ± 0.10 nmol/mg, *p* < 0.05) (Figures 6A,B). Overexpression of SLPI by AAV9-SLPI could significantly reduce PC content level when compared with AAV9 group (tissue: 0.69 ± 0.09 nmol/mg vs. 1.186 ± 0.14 nmol/mg, *p* < 0.05, serum: 0.80 ± 0.23 nmol/mg vs. 1.44 ± 0.73 nmol/mg, *p* < 0.05) (Figures 6A,B).

Similarly, the results showed that both serum and cardiac tissue IMA levels in AAV9 group subjected to I/R injury were significantly higher when compared with sham group (tissue: 0.25 ± 0.04 A.U. vs. 0.07 ± 0.01 A.U., *p* < 0.05, serum: 0.24 ± 0.03 arbitrary unit (A.U.) vs. 0.18 ± 0.02 A.U. *p* < 0.05) (Figures 6C,D). Overexpression of SLPI by AAV9-SLPI could significantly reduce serum and cardiac tissue IMA levels when compared with AAV9 group (tissue: 0.14 ± 0.01 A.U. vs. 0.25 ± 0.04 A.U., *p* < 0.05, serum: 0.19 ± 0.02 A.U. vs. 0.24 ± 0.03 A.U., *p* < 0.05) (Figures 6C,D).

## Discussion

**Figure 7:**
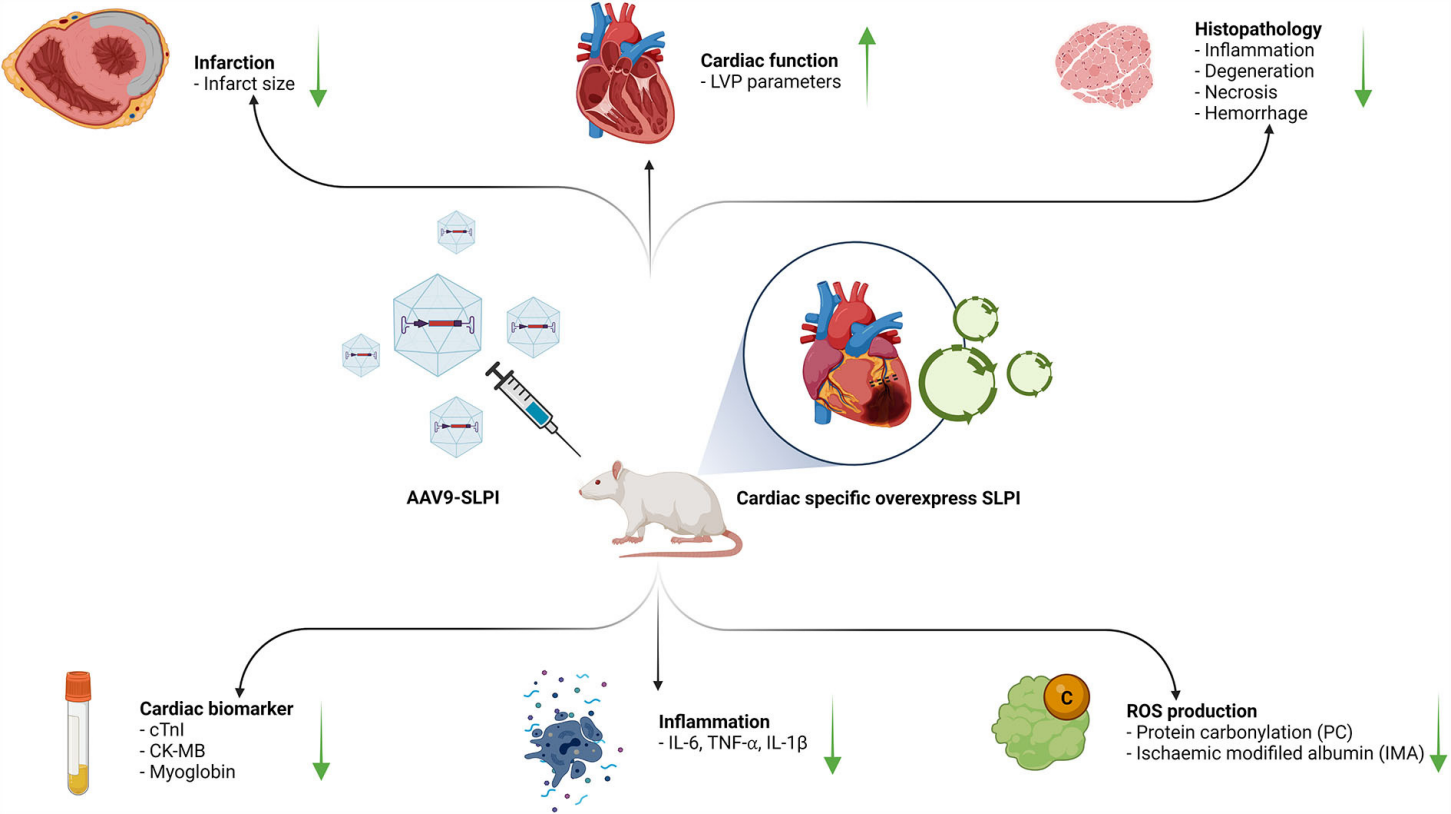
A schematic diagram of the major findings in this study. The cardiac selective overexpression of SLPI by using AAV9-SLPI. Overexpression SLPI could provide the cardioprotective effect associated with the attenuation of inflammation, intracellular ROS production related to reducing infarct size and cardiac biomarkers and improving cardiac function. This figure generated by BioRender with access URL https://app.Biorender.com/illustrations/62a4bc9045f21a4fdcc51874.

This study elucidated the cardioprotective effect of secretory leukocyte protease inhibitor against myocardial I/R injury by means of gene delivery for SLPI-selective overexpression in cardiac tissue. The adeno-associated virus serotype 9 (AAV9) delivered human SLPI cDNA and showed a protective effect against ischemia/reperfusion (I/R) injury by reducing infarct size and inflammatory cytokines through oxidatively modifying protein carbonyl content, ischemia-modified albumin, and cardiac injury markers, thereby improving the cardiac function (Figure 7).

Protease enzymes play a major role in maintaining the protein structure of cellular homeostasis in normal conditions. An imbalance of protease enzyme activity and its inhibitor causes cellular protein lysis, consequently leading to cellular injury (29). Upregulation of protease enzyme activity occurs during myocardial ischemia/reperfusion (30). During ischemia, a lack of blood supply leads to anaerobic metabolism that induces intracellular acidosis and ATP depletion (31). Reactive oxygen species (ROS) increase, with neutrophil accumulation in the ischemic area (32). Accumulation of neutrophils upregulates the inflammatory response and protease enzyme level, especially serine protease enzyme that is involved in cardiac injury together with calpain, cathepsin, matrix metalloproteinases (MMPs), and chymase (33). Alterations in protease activity cause cardiac cell injury and death and also result in subcellular cardiac remodeling and loss of cardiac functions. Among these proteases, chymase contributes to the process of cardiac remodeling and heart failure (34). Therefore, inhibition of protease activity can be a powerful strategy to reduce cellular injury by preventing the expansion of injured tissue and also the progression of cardiac hypertrophic remodeling, ultimately reducing patient morbidity and mortality.

In several mice and dog experimental I/R models, a protease enzyme inhibitor application reduced cell injury and infarct size (35, 36). Our previous studies on recombinant human secretory leukocyte protease inhibitor (rhSLPI) administration showed reduced cell injury and cell death in both *in vitro* and *ex vivo* I/R models (4–7). SLPI reduced ROS levels and inflammatory cytokine production (12–15). Moreover, SLPI downregulated the p38 MAPK phosphorylation and apoptotic pathway. These results indicate that protease enzyme inhibitor SLPI has potential therapeutic effects in myocardial I/R injury from adverse mechanisms (12–14). SLPI shows a high expression of beneficial effects in mucosal tissues, but a low expression in cardiac tissues. Manipulation of gene expression to elucidate the beneficial effect on the heart appears challenging. In an *in vitro* experiment, overexpression of the *slpi* gene was performed in the H9c2 cell line. The ectopic expression of rhSLPI in cardiac cells provides useful information, but overexpression of SLPI in H9c2 cells may not be closely related to real physiological settings in the intact heart. Therefore, overexpression of SLPI in the intact heart will provide more functional data related to actual cardiac physiology and provide more reliable interpretations.

Reactive oxygen species (ROS) are regarded as detrimental factors from I/R injury that burst during reperfusion. Abnormal elevation of ROS induces biomolecular modification of DNA, lipids, and in particular proteins in the body (37). This results in damaged cell structure and inflammatory cell infiltration into the injury area that aggravates cell death (38). SLPI reduced the intracellular ROS level in ischemia/reperfusion injury in cardiomyocyte and endothelial cell experiments (13, 14), while products of oxidative stress including protein carbonyl (PC) and ischemia-modified albumin (IMA) were used as oxidative stress markers in patients with myocardial ischemia after percutaneous coronary intervention and left ventricular hypertrophy (39, 40). In this study, cardiac-selective SLPI expression reduced protein carbonyl content level and ischemia-modified albumin in both cardiac tissue and serum (Figure 6), with reduced infarct size and improved cardiac function. These results concurred with those of our previous study of SLPI intravenous post-ischemic treatment in an *in vivo* I/R injury model (15). Another deleterious impact of I/R injury is the overproduction of proinflammatory cytokines that promotes recruitment and activation of neutrophils infiltrated into the myocardium and extends the injury area. As demonstrated in our earlier study, SLPI reduced proinflammatory cytokine levels including IL-6, TNF-α, and IL-1β in post-ischemic treatment of I/R injury (15). In this study, cardiac-selective expression of hSLPI reduced inflammatory cytokines in a similar manner to intravenous administration including IL-6, TNF-α, and IL-1β in both blood serum and cardiac tissue extraction (Figure 4). Therefore, the biological protection mechanisms of SLPI against myocardial I/R injury are mediated through the attenuation of ROS and proinflammatory cytokines as key molecules involved in the progression of tissue inflammation and injury. In 2008, Schneeberger reported that cardiac transplant tissue preserved in an SLPI solution showed less severe necrosis and inflammation compared with graft tissue of SLPI^−/−^ mice (41). SLPI preservative solution also lowered cell detachment, elastic membrane disruption, necrosis, and degeneration in aortic ring histopathology (42). Our study showed that cardiac-selective overexpression of hSLPI using AAV9-SLPI reduced inflammation, degeneration, necrosis, and hemorrhage in the histopathology of cardiac tissue (Figure 5).

Efficient delivery of foreign DNA into cardiomyocytes is crucial for cardiac gene overexpression research. Several plasmid transfection methods show very low transfection efficiencies in heart tissue (43). Achieving efficient *in vivo* gene delivery to cardiac tissue is difficult compared with other postmitotic tissues such as the brain and retina. To overcome this difficulty, a non-pathogenic human parvovirus, adeno-associated virus (AAV), has been studied and used for gene delivery (44, 45). AAV serotype 9 (AAV9) showed high-efficiency transduction in cardiac cells compared with other serotypes (46). Therefore, in this study, AAV9-SLPI was customized with cardiac-selective expression under the control of the cardiac troponin (cTn) promoter. The dosage in this study at 0.5 × 10^12^ viral particles per animal was lower than in previous studies (47–49), but a high expression of both protein and gene in myocardial tissue was recorded (Figure 2), suggesting that AAV9 provided efficient gene transduction in the heart. Determination of systemic injury or toxicity by measuring the kidney and hepatic biomarkers demonstrated the safety of AAV9 transfection as well as SLPI overexpression (Figure 1). The reduction in cardiac injury markers including cTnI, CK-MB, and myoglobin level was related to reduced infarct size, which was also demonstrated in rats receiving AAV9-SLPI (Figure 3). This reduction in cardiac injury markers suggested an improvement in cardiac function of the treated group (Table 1). Our data indicated that cardiac-selective overexpression of SLPI by AAV9 delivery provided cardioprotection against myocardial I/R injury.

Several forms of cardiac cell death and related molecular mechanisms have been previously discussed. Intracellular ROS induced DNA damage, leading to apoptosis, necroptosis by inflammatory cytokine signaling activation, or pyroptosis due to inflammasome activation (50). Our previous findings showed the cardioprotective effect of SLPI *via* the reduction of intracellular ROS production, activation of Akt survival kinase, and inhibition of the p38 MAPK pathway that plays a major role in I/R injury by reducing inflammation and also attenuating apoptosis (12–15, 42). Overexpression of the SLPI gene by AAV9 gene delivery might involve several protective pathways. SLPI may precondition the heart by activating the Akt pro-survival pathway, reducing p38 MAPK, and attenuating apoptosis. Alternatively, the anti-inflammatory effect of SLPI might reduce inflammatory cytokine production, thereby aggravating necroptosis. SLPI dose-dependently inhibited ATP-mediated inflammasome activation and IL-1β release (51) and may be involved in the attenuation of NLRP3 inflammasome activation related to autophagy in myocardial I/R injury (52). However, the actual mechanisms and cardioprotective pathways require further intensive investigation.

This study has several limitations. The current study did not measure the cardiac inflammatory protease activity, which could be useful to elucidate whether the cardioprotection is really due to the inhibition of protease activity by the secretion of SLPI from cardiomyocytes. Furthermore, overexpression of SLPI significantly increased heart weight, although the body weight of the animal was also increased due to difference in animal age. However, increasing heart weight could possibly cause a serious concern about safety of long-term SLPI expression on hypertrophy, although histopathological assessment could not detect any hypertrophic appearances and fibrosis, and there was no significance of the mean cardiomyocyte cross-sectional area among groups (data not shown). Therefore, echocardiography assessment before and after AAV treatment and histopathological assessment on cardiac remodeling should be considered as another safety information. Basal cardiac function of non-injured heart from animals after receiving AAV9 or AAV9-SLPI to elucidate ectopic gene expression of SLPI in cardiac tissue alter cardiac function was not conducted, while the only parameters demonstrating no cardiotoxicity of cardiac-selective gene expression of SLPI were cardiac injury markers (Figures 1H–J). Additional cardiac histopathological information such as quantitative assessment of apoptotic cardiomyocytes (TUNEL assay) and inflammatory cell infiltration was also not included. SLPI provided cardioprotection against I/R injury, but several concerns remain. SLPI promotes metastasis in some cancer types (53), while long-term administration of serine protease inhibitor may develop metabolic side effects (54) and spontaneous bleeding in hemophiliac patients (55). This study did not demonstrate the stability of gene expression by AAV9 delivery. Further studies are required to assess the stability of SLPI gene expression and requirements for repeat injections as well as the long-term side effects. Only healthy adult male rats were used in our experiments, while the study protocol determined only the acute effect of SLPI expression. The results, therefore, may not be suitable for clinical translation. Further studies following *in vivo* step-by-step criteria for **IM**proving **P**reclinical **A**ssessment of **C**ardioprotective Therapies (“IMPACT”) (56) using different sex, age, and larger animal models are necessary to evaluate the long-term effects of chronic toxicity, hypertrophy, mortality, and/or heart failure as endpoints, as well as the risks of developing malignancies from gene therapy to provide essential basic data for further clinical trials.

## Conclusion

Our findings show for the first time that adeno-associated virus gene delivery of cardiac-selective expression of SLPI provided cardioprotection by reducing infarct size, inflammatory cytokines, and ROS production and improving cardiac function against *in vivo* myocardial I/R injury. Overexpression of the protease inhibitor using cardiac-selective overexpression of SLPI was shown to be a novel modality and be fundamental evidence for alternative therapeutic strategy for ischemic heart disease.

## Data availability statement

The datasets presented in this study can be found in online repositories. The names of the repository and accession number(s) can be found below: https://www.ncbi.nlm.nih.gov/genbank, NM_003055.

## Ethics statement

The animal study was reviewed and approved by All care and use of the animal subjects followed the guidelines of the ARRIVE guidelines and the guidance on the operation of the animals (Scientific Procedures) Act 1986 and the World Health Organization Guidelines for Breeding and Care of Laboratory Animals. All animal protocols were approved by the Institutional Animal Care and Use Committee, Chulalongkorn University Laboratory Animal Centre (CULAC, Protocol.1973025). The experiments were followed by animal research guideline to minimize the pain, suffering and distress.

## Author contributions

SK, PM, NN, and AK conceived and designed the experiments. PM, AK, NN, FP, WC, RY, SU, and SK performed the experiments and analyzed the data. SK, AK, NN, and SU contributed reagents/materials/analytical tools. All authors wrote, read, prepared, and approved the manuscript.

## Funding

This work was supported by the National Research Council of Thailand (NRCT) in cooperation with Naresuan University (Grant No. R2564B013) for NN and SK. This project was funded by the National Research Council of Thailand (NRCT) and Chiang Mai University: N42A650305 for SK and Chiang Mai University TA/RA scholarship for FP and WC.

## Conflict of interest

The authors declare that the research was conducted in the absence of any commercial or financial relationships that could be construed as a potential conflict of interest.

## Publisher's note

All claims expressed in this article are solely those of the authors and do not necessarily represent those of their affiliated organizations, or those of the publisher, the editors and the reviewers. Any product that may be evaluated in this article, or claim that may be made by its manufacturer, is not guaranteed or endorsed by the publisher.
